# BACH transcription factors in diseases of the exocrine and endocrine pancreas: effects, mechanisms, and clinical implications

**DOI:** 10.3389/fmed.2026.1805552

**Published:** 2026-04-22

**Authors:** Robert Jaster

**Affiliations:** Division of Gastroenterology, Hepatology and Nutritional Medicine, Department of Internal Medicine, Rostock University Medical Center, Rostock, Germany

**Keywords:** BACH1, BACH2, diabetes mellitus, pancreatic cancer, pancreatitis

## Abstract

The BTB and CNC homology (BACH) proteins BACH1 and BACH2 are highly conserved transcriptional repressors that exert their effects by forming heterodimers with small musculoaponeurotic fibrosarcoma (MAF) proteins. BACH1 is ubiquitously expressed and functions by competing with nuclear factor erythroid 2-like 2 (NRF2) for binding to MAF. By suppressing the transcription of heme oxygenase 1 (*HO-1*/*HMOX1*), BACH1 inhibits the expression of genes involved in cellular oxidative stress responses. Expression of BACH2 is largely restricted to immune cells in both innate and adaptive compartments, where it plays a central role in hematopoietic development and immune cell differentiation. By engaging stretch or super-enhancers, BACH2 targets gene networks that include cytokines and cytokine receptors. Both BACH proteins have been implicated in numerous chronic diseases, including cancer and immune-mediated chronic inflammatory diseases. Aberrant activation is a key driver of BACH1-related pathogenesis, whereas BACH2 is typically linked to loss-of-function alterations. This article reviews the role of BACH proteins in diseases of the endocrine and exocrine pancreas, with a focus on type 1 and 2 diabetes mellitus, pancreatitis, and pancreatic cancer. Mechanistic aspects, pathophysiological correlations, and potential avenues for targeted therapies are discussed.

## 1 Introduction

The BTB and CNC homology (BACH) proteins BACH1 and BACH2 are highly conserved transcriptional repressors that belong to the cap “n” collar/basic leucine zipper (bZIP) family of transcription factors. Through their bZIP domain, BACH proteins form heterodimers with small musculoaponeurotic fibrosarcoma (MAF) proteins, while BACH2 additionally interacts with basic leucine zipper ATF-like transcription factor (BATF) family members (1, 2).

BACH1 is ubiquitously expressed and functions as a key antagonist of nuclear factor erythroid 2-like 2 (NRF2) by competing for MAF binding. As a result, BACH1 suppresses the transcription of heme oxygenase 1 (*HO-1/HMOX1*) and a broad range of genes involved in cellular oxidative stress responses (3). Consistent with this repressive role, aberrant BACH1 activity has been implicated in the pathogenesis of multiple chronic diseases, including cancer—where it drives tumor cell proliferation and metastatic progression—as well as neurodegenerative disorders, chronic inflammatory bowel disease, pulmonary fibrosis, and inflammatory skin diseases (1).

In contrast to the widespread expression of BACH1, BACH2 is largely restricted to immune cells in both innate and adaptive compartments, as well as to neuronal cells. BACH2 plays a central role in hematopoietic development and immune cell differentiation, particularly in lineage commitment and the maturation of innate and adaptive immune populations [reviewed in (2)]. At the molecular level, BACH2 orchestrates immune cell activation by regulating extensive gene networks that include numerous cytokines and cytokine receptors, primarily through the engagement of stretch or super-enhancers (4). BACH2 has been shown to influence immunological phenotypes such as lymphocyte, monocyte, and neutrophil counts in peripheral blood (5), in addition to IgG glycosylation (6). Genetic and functional studies have implicated BACH2 in the pathogenesis of multiple autoimmune and immune-mediated chronic inflammatory diseases, including Crohn's disease (7), celiac disease (8), and multiple sclerosis (9). Furthermore, *BACH2* haploinsufficiency may result in a syndrome characterized by immunodeficiency and autoimmune disorders (4).

Diseases of the endocrine and exocrine pancreas are among the most common diseases worldwide. Recent publications on BACH proteins have provided evidence of physiological and pathophysiological links to both the endocrine and exocrine pancreatic compartments. This review, therefore, examines the role of BACH proteins across compartments and focuses on three particularly important diseases. Firstly, a chapter on the endocrine pancreas covers diabetes mellitus, including type 1 (T1D) and type 2 (T2D). Type 1 diabetes (T1D) is characterized by autoimmune destruction of pancreatic β-cells, leading to insulin deficiency, whereas the much more common type 2 diabetes (T2D) primarily results from insulin resistance combined with relative insulin deficiency (10). Secondly, a chapter on the endocrine pancreas addresses pancreatitis and pancreatic carcinoma. Acute pancreatitis (AP) and chronic pancreatitis (CP) are among the most common causes of gastrointestinal-related office visits and hospital admissions (11). Pancreatic ductal adenocarcinoma (PDAC), the most common form of pancreatic carcinoma, has a 5-year survival rate as low as 10%. Accordingly, PDAC is one of the most lethal cancers worldwide, and its incidence is increasing (12).

## 2 Endocrine pancreas

Given *BACH1's* important role in responses to metabolic and oxidative stress, it is reasonable to ask about its possible role in T2D. Studies in mice by Kondo et al. (13) showed that *BACH1* deficiency significantly suppressed alloxan-induced reduction in pancreatic insulin content and the resultant glucose elevation. A key mechanism here is the upregulation of *HO-1* expression in islets of *BACH1* knockout mice, which protects pancreatic β-cells from oxidative stress-induced apoptosis (13). Studies on the mechanisms of lipotoxicity in β-cells revealed that, in addition to LXR, PPARα, and FOXO1, BACH1 is a key transcription factor orchestrating the metabolic and oxidative stress responses to palmitate (14). Interestingly, hepatic BACH1 knockdown ameliorates hyperglycemia and improves insulin sensitivity in diabetic mice. This appears to be due to reduced interaction between the BACH1-binding partner, protein tyrosine phosphatase 1B, and the insulin receptor β (IR-β) in BACH1 knockdown cells (15). On the other hand, *BACH1* deficiency in mice with a global (rather than hepatocyte-specific) knockout only minimally impacted obesity and insulin resistance after high-fat diet loading (13). Differences in diet composition and between the respective models could explain these discrepant observations. Taken together, available data suggest, albeit not unequivocally, that *BACH1* plays critical roles in oxidative stress in pancreatic β-cells, in the regulation of insulin signaling, and in glucose homeostasis.

Like *BACH1, BACH2* appears to contribute unfavorably to T2M, although the molecular mechanisms remain to be studied. Expression of BACH2 was found to be positively correlated with the ratio of β-cell dedifferentiation in humans (16). Knocking out *BACH2* in T2D islets reversed the cellular features of the disease and restored a non-diabetic phenotype, while treatment with a BACH inhibitor increased insulin secretion (17). However, for both BACH proteins, human studies and mechanistic insights are still limited, so it is currently unclear whether inhibiting either could be a useful therapeutic approach for T2D.

The situation is fundamentally different in autoimmune diabetes (T1D), where *BACH2* is ascribed an important protective function. First strong evidence for a role of *BACH2* in T1D came from genetic studies. In a 2008 meta-analysis of genome-wide association study data, Cooper et al. (18) identified a T1D risk locus on chromosome 6q15 that contained *BACH2* as the only gene. The most associated SNP in this study, rs11755527, is located in intron 3 of *BACH2*. Later, large-scale genome-wide association studies and meta-analyses confirmed the strong association of rs11755527 with T1D (19) and reported the same for the intronic SNP rs3757247, which is in tight linkage disequilibrium with rs11755527 (20). There are divergent findings across cohorts from Poland (21) and southern Brazil (22), which may be due to small sample sizes or ethnic differences. A recent fine mapping of the *BACH2* locus refined the T1D association to the two intronic variants, rs72928038 and rs6908626 (23). As the mechanistic explanation, the authors suggest that rs72928038:G>A, the T1D-associated allele, disrupts binding of the transcription factor ETS1 at an enhancer that promotes BACH2 expression in naïve CD4+ T cells.

Further insights into the role of BACH2 in T1D were gained by *in vitro* studies using human islets and human or rodent cell lines. Specifically, BACH2 inhibition exacerbated cytokine-induced β-cell apoptosis via the mitochondrial pathway of cell death, whereas BACH2 overexpression exerted protective effects (24, 25). The effects of BACH2 are mediated by crosstalk with another T1D candidate gene, tyrosine-protein phosphatase non-receptor type 2 (*PTPN2*), and joint regulation of the JNK1/BIM and STAT1 pathways (24, 26). Mechanistically, BACH2 induces the expression of PTPN2, which mediates the dephosphorylation of STAT1 at the tyrosine residue Y701 (direct effect) and at the serine residue S727 (indirectly via JNK1 and p38 MAPK) (26). This establishes a negative feedback loop for IFN-α signaling in β-cells, preventing the overexpression of proinflammatory chemokines (e.g., CXCL10) and of antigen presentation via the major histocompatibility complex HLA. In addition, BACH2 upregulates Gadd45b, a Mitogen-Activated Protein Kinase Kinase 7 (MKK7) inhibitor, in β-cells, thereby limiting IL-1β + IFN-γ-induced activation of the MKK7-JNK1 pathway and, ultimately, reducing pancreatic β-cell apoptosis (24).

Of particular interest, a recent study identified a distinct subset of CD4^+^ T cells specifically present in pancreatic lymph nodes of organ donors representing the active T1D disease stage. These T cells exhibited elevated activity of *NFKB1* and *BACH2*, along with extensive chromatin remodeling associated with these transcription factors, and might correspond to the autoimmune progenitor pool. The authors hypothesize that BACH2 may suppress cytokine expression within this progenitor compartment (27).

**Key Insights:** Both BACH proteins are implicated in unfavorable roles in T2D, whereas BACH2 appears to be protective in T1D.

## 3 Exocrine pancreas

### 3.1 Pancreatitis

The molecular determinants of the transition from mild AP to a severe, necrotizing form with high mortality are still largely unknown. A recent study by Zhou et al. showed that BACH1 levels were significantly increased in patients with severe AP and in cellular and animal models of the disease. Experimental depletion of BACH1 reduced oxidative stress, ferroptosis, and inflammatory responses, thereby improving cell survival. The detrimental effects of BACH1 appear to be mediated, at least in part, by heat shock protein B1 (HSPB1) (28).

In CP, recurrent episodes of inflammation lead to progressive destruction of the pancreatic parenchyma, ultimately replaced by fibrotic connective tissue. Data from Sasikala et al. suggest a protective role for BACH2 in this process: specifically, the authors deciphered transcriptional repression of the *BACH2* gene in CD4^+^ T lymphocytes of CP patients as a potential key event driving polarization of pathogenic Th17 cells. Furthermore, they identified a novel *BACH2* gene variant (SNP rs9111 in the 5′-UTR) that, in individuals with primary genetic susceptibility to CP, was associated with advanced disease (29). In autoimmune pancreatitis (AIP), a rare variant of CP, BACH2 may also serve as a protective factor that counteracts disease progression. Data from our own laboratory show that *BACH2*-deficient mice are prone to spontaneous AIP, most likely due to disturbed immune homeostasis and dysregulated adaptive immune activation (30, 31).

**Key Insights:** Recent literature indicates opposing roles for BACH1 and BACH2, with BACH1 aggravating AP and BACH2 protecting against CP.

### 3.2 Pancreatic ductal adenocarcinoma

Recent studies have implicated *BACH1* in various processes of tumorigenesis and tumor progression, including cancer metabolism, metastasis, proliferation, and chemoresistance (32). There are also a number of publications on PDAC, though they do not yet provide a consistent overall picture.

In 2011, Wu et al. (33) identified five loci associated with pancreatic cancer susceptibility in Chinese populations through a genome-wide association study. The strongest association signal was the rs372883T>C variation located in the 3′-untranslated region (3′UTR) of *BACH1* on 21q21.3. People with the T allele had a significantly higher risk of PDAC than those with the C allele. In a follow-up study, Huang et al. (34) showed that the rs372883T allele was associated with significantly lower BACH1 levels than the rs372883C allele in both tumor and normal tissues. Moreover, PDAC patients with the rs372883T allele were more resistant to gemcitabine and had shorter survival than those with the rs372883C allele. Further evidence for a potential tumor *suppressor* role of BACH1 in PDAC came from accompanying cell biological and molecular studies. They showed that knockdown of BACH1 expression stimulated PDAC cell proliferation and angiogenesis and favored the expression of markers of epithelial-to-mesenchymal transition (EMT), whereas BACH1 overexpression had the opposite effect (34). BACH1, in complex with the small MAF protein MAfF, has recently also been implicated in the global epigenetic response of pancreatic cancer cells to gemcitabine. Gemcitabine-induced translocation of protein arginine methyltransferase 1 (PRMT1) limits the assembly of chromatin-bound MAfF/BACH1 transcriptional complexes, thereby promoting chemoresistance (35).

The data on protective effects contrast with other recent studies that strongly support a *protumorigenic* role for BACH1 in PDAC. By analyzing BACH1 mRNA and protein expression in human PDAC samples, Sato et al. (36) identified high BACH1 expression as a *poor* prognostic factor. Accompanying studies investigating human PDAC cell lines *in vitro* and orthotopic cell implants in mice suggested that BACH1 also promotes pancreatic cancer metastasis by repressing epithelial genes and inducing EMT. As a key mechanism, repression of iron metabolism-related genes in PDAC cells has emerged, leading to increased labile iron and subsequent suppression of E-cadherin expression (37, 38). An inverse correlation between BACH1 expression and the prognosis of PDAC patients was also reported by Kim et al. (39).

How can these sometimes contradictory observations be reconciled? First of all, the overall data situation remains too sparse and heterogeneous to draw final conclusions in this regard. The studies on cell cultures should be extended to models closer to the patient, such as patient-derived organoids from resectates or punctates. At the animal model level, there is a need for studies on genetically engineered mouse models of PDAC, which currently best reflect human pathogenesis. Prospective clinical studies are needed to better assess the prognostic value of BACH1 as a biomarker of survival or treatment response. From a mechanistic perspective, it is of particular interest to understand how BACH1 integrates into the established intra- and intercellular molecular networks in PDAC. Last but not least, the effects of BACH1 on PDAC progression may depend on the clinical and pathological stage, the molecular subtype (40), the specific microenvironment, and the patient's immune status. All these aspects should be taken into account in future studies. This could also provide insights into whether BACH1 itself could possibly be considered as a therapeutic target in PDAC.

To date, no studies have examined the potential role of BACH2 in PDAC; nevertheless, investigating this may be worthwhile: a growing number of oncological studies suggest that BACH2 is involved not only in hematological malignancies but also in solid organ tumors at multiple levels. The mechanisms of BACH2 action include immunosuppressive effects, modulation of chemosensitivity and resistance, and modulation of the tumor immune microenvironment (and thus processes of invasion and metastasis).

**Key Insights:** Recent data implicate BACH1 in the pathogenesis and progression of PDAC. Apparent contradictions among individual findings may reflect the heterogeneity of the available data.

## 3 Discussion

There is increasing evidence that BACH proteins are significantly involved in presumably all major diseases of the endocrine and exocrine pancreas, in particular diabetes mellitus, pancreatitis, and pancreatic cancer.

In T2D, the most common disease of the endocrine pancreas, pathogenic roles have been proposed for both BACH proteins (13–17). In contrast, in autoimmune diabetes, BACH2 exerts protective effects: Gene variants that are suspected of affecting BACH2 expression or function are associated with T1D (18–20, 23), and expression of BACH2 supports the survival of pancreatic β-cells in response to pro-apoptotic stimuli (24–26). These observations are consistent with the known immunoregulatory action of the transcriptional repressor but may also reflect incompletely characterized local effects in islet cells.

With regard to the exocrine pancreas, links between BACH1 and PDAC are increasingly being reported (33–39). In current studies, the evidence for an aggravating or tumor-promoting effect predominates, but the picture is not uniform. The course of AP could also be unfavorably influenced by BACH1 (28). BACH1 actions are at least partially related to its molecular functions in oxidative stress response, cell cycle regulation, and metabolic homeostasis (especially heme). As in the endocrine compartment, BACH2 also counteracts inflammatory and autoimmune processes in the exocrine pancreas (29, 30).

**Figure 1:**
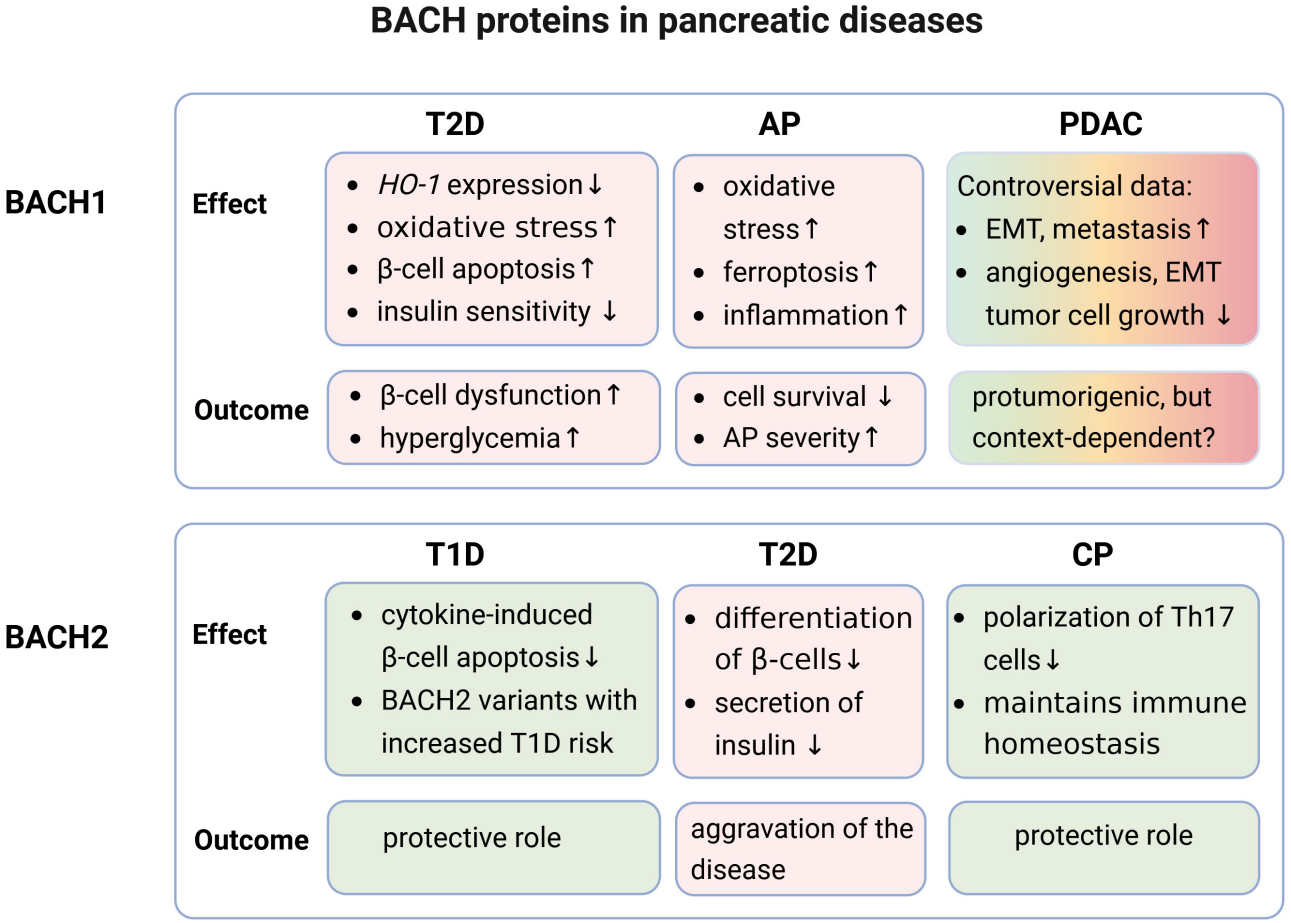
Role of BACH proteins in pancreatic diseases. The effects of BACH1 and BACH2 in diabetes mellitus, pancreatitis, and pancreatic cancer, as outlined in this review, are summarized and evaluated with respect to their influence on the corresponding diseases. Protective and unfavorable effects are highlighted in light green and light red, respectively. T1D, diabetes mellitus type 1; T2D, diabetes mellitus type 2; AP, acute pancreatitis; CP, chronic pancreatitis; PDAC, pancreatic ductal adenocarcinoma, HO-1, heme oxygenase 1; EMT, epithelial-to-mesenchymal transition. Created in BioRender. Jaster, R. (2026) https://BioRender.com/db2sd7o.

Figure 1 provides an overview of the key findings regarding the role of BACH proteins in pancreatic diseases. The genetic associations are summarized in Table 1.

**Table 1:** Gene variants of *BACH1* and *BACH2* associated with pancreatic diseases.

SNP	Gene	Localization	Association	Molecular effect	References
rs372883	BACH1	3′-UTR	PDAC	might affect interaction of BACH1 with microRNAs and alter its expression	(33)
rs11755527	BACH2	intronic	T1D	unknown	(18) (19)
rs3757247	BACH2	intronic	T1D	unknown	(19) (20)
rs72928038	BACH2	intronic	T1D	disrupts ETS1 binding at a BACH2 enhancer in naïve CD4? T cells	(23)
rs6908626	BACH2	intronic	T1D	unknown	(23)
rs9111	BACH2	5′-UTR	CP	Lower expression of BACH2	(29)

SNP, single nucleotide polymorphism; UTR, untranslated region; T1D, diabetes mellitus type 1; T2D, diabetes mellitus type 2; CP, chronic pancreatitis; PDAC, pancreatic ductal adenocarcinoma.

It is conceivable that BACH proteins could become therapeutic targets for diabetes mellitus, pancreatitis, and PDAC in the future. A first experimental study used compound 8 (41), a substituted fused imidazole derivative that inhibits both BACH1 and BACH2 (17). The compound reduced blood glucose levels and increased plasma insulin in diabetic mice. Additionally, it restored insulin secretion in both diabetic mice and human islets (17). However, beyond the current lack of clinically applicable BACH agonists and antagonists, there are still hurdles to overcome. These have already been outlined in the previous sections. In particular, it is necessary to expand mechanistic understanding of BACH proteins in a context- and cell-type-specific manner. For example, while the role of BACH2 in immune cells is relatively well characterized, its functions in pancreatic parenchymal cells are less well understood; consequently, non-selective inhibition of this transcription factor could also lead to undesirable effects. What gives hope is that transcription factors, once deemed “undruggable,” are now increasingly targetable, and several promising therapeutic strategies are emerging in cancer and beyond. These approaches include, but are not limited to, targeting protein–protein interactions and the DNA-binding activity of transcription factors, as well as indirect strategies such as modulating upstream kinases or epigenetic regulation, and promoting transcription factor degradation (42).
